# Using ROC Curves to Choose Minimally Important Change Thresholds when Sensitivity and Specificity Are Valued Equally: The Forgotten Lesson of Pythagoras. Theoretical Considerations and an Example Application of Change in Health Status

**DOI:** 10.1371/journal.pone.0114468

**Published:** 2014-12-04

**Authors:** Robert Froud, Gary Abel

**Affiliations:** 1 Clinical Trials Unit, Warwick Medical School, University of Warwick, Gibbet Hill Road, Coventry, United Kingdom; 2 Norge Helsehøyskole, Campus Kristiania, Prinsens Gate 7–9, Oslo, Norway; 3 Cambridge Centre for Health Services Research, University of Cambridge, Robinson Way Cambridge, Cambridgeshire, United Kingdom; Public Health Agency of Barcelona, Spain

## Abstract

**Background:**

Receiver Operator Characteristic (ROC) curves are being used to identify Minimally Important Change (MIC) thresholds on scales that measure a change in health status. In quasi-continuous patient reported outcome measures, such as those that measure changes in chronic diseases with variable clinical trajectories, sensitivity and specificity are often valued equally. Notwithstanding methodologists agreeing that these should be valued equally, different approaches have been taken to estimating MIC thresholds using ROC curves.

**Aims and objectives:**

We aimed to compare the different approaches used with a new approach, exploring the extent to which the methods choose different thresholds, and considering the effect of differences on conclusions in responder analyses.

**Methods:**

Using graphical methods, hypothetical data, and data from a large randomised controlled trial of manual therapy for low back pain, we compared two existing approaches with a new approach that is based on the addition of the sums of squares of 1-sensitivity and 1-specificity.

**Results:**

There can be divergence in the thresholds chosen by different estimators. The cut-point selected by different estimators is dependent on the relationship between the cut-points in ROC space and the different contours described by the estimators. In particular, asymmetry and the number of possible cut-points affects threshold selection.

**Conclusion:**

Choice of MIC estimator is important. Different methods for choosing cut-points can lead to materially different MIC thresholds and thus affect results of responder analyses and trial conclusions. An estimator based on the smallest sum of squares of 1-sensitivity and 1-specificity is preferable when sensitivity and specificity are valued equally. Unlike other methods currently in use, the cut-point chosen by the sum of squares method always and efficiently chooses the cut-point closest to the top-left corner of ROC space, regardless of the shape of the ROC curve.

## Introduction

Initially developed during World War II for use in interpreting radar signals, receiver operator characteristic (ROC) curves are commonly used in medical research to evaluate screening tests, identify thresholds to facilitate decision-making about patients, and to quantify the responsiveness of quasi-continuous patient-reported outcomes measures (PROMs). [1], [2] Our focus in this paper is on their use to estimate minimally important change (MIC) thresholds on PROMs. [3]

MIC is usually defined as smallest magnitude of change that can be considered important (at the level of the individual), and in the absence of troublesome side-effects and excessive costs, mandates a change in a patient's management. [4], [5] In 1986, Deyo and Centor suggested that PROMs could be viewed as diagnostic tests; in the sense that they can be thought to be diagnostic of improvement. [6] Using an external criterion – for example a health transition question or an accepted gold standard diagnostic test for improvement – continuous, or quasi-continuous, PROM change scores can be plotted on a ROC curve, facilitating a choice of cut-point associated with optimal sensitivity and specificity. [6] When estimating MIC in chronic conditions with variable clinical trajectories, such as low back pain, sensitivity and specificity are assumed to be of equal value. [7]–[10]

Epidemiologists have taken different approaches to calculating the optimum MIC cut-point using ROC curves even though they agree that sensitivity and specificity should be valued equally. [7], [11], [12] For example, Farrar *et al*, [12] choose the point closest to the intersection of a −45° tangent line (*i.e.* passing from (0,1) to (1,0), intersecting the ROC curve). As Farrar points out, mathematically this is equivalent to the point at which sensitivity and specificity are closest together. [12] In contrast, an approach by researchers from the EMGO institute, is to choose the point closest to the top-left corner of the ROC curve, which they suggest is found by choosing the cut-point that minimises the sum of 1 – sensitivity and 1 – specificity. [7], [13] The Farrar and EMGO approaches can often produce points that differ, and this may lead to different conclusions about responder thresholds and results of responder analyses. We suggest that a third approach may be optimal and preferable. We assert that the commonly practised EMGO method does not in general select the point closest to the top-left corner of the ROC curve, and that the 1 – sensitivity and 1 – specificity terms should first be squared before summing, in accordance with Pythagoras' theorem. In this paper, we first present contour diagrams highlighting the nature of the divergence of these three different approaches, and then using both hypothetical and real trial data we explore the extent of the possible divergence between these approaches.

## Methods

Equations 1, 2, and 3 show the different estimators being considered. In all cases we choose the point in ROC space that minimises the quantity 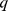, where 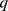 is defined differently depending on which estimator is used. In equation 1, the Farrar method is used to estimate 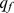 by minimising the gap between sensitivity (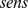) and specificity (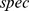) for all possible cut-points on the instrument, that are contained within ROC space. In equation 2, the EMGO method is used to estimate 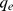 by minimising the sum of 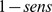 and 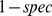. Equation 3 is the method we propose, which estimates 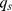 by minimising the sums of squares of 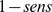 and 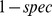, and which, as follows from simple Euclidean geometry, is closest to the top-left corner (1,0) of ROC space.
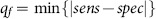
(1)

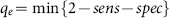
(2)

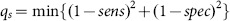
(3)


For each of these estimators, we first constructed diagrams showing contours of the quantities being minimised, to illustrate the how methods favour different areas of ROC space. Using these diagrams we illustrated how the three different methods can give rise to similar or different estimates of MIC in the situation where the number of possible cut-points is large, *i.e.* tending toward a truly continuous nature. Next, this was done using three hypothetical examples of well-behaved ROC curves, by which we mean the second derivative of the curve is always negative (*i.e* the gradient is always decreasing, moving from left to right). And finally, we selected real examples using data from the UK Back pain Exercise And Manipulation (UK BEAM) trial to explore the extent of differences in practice, where ROC curves are seldom well-behaved. [14]

### The UK BEAM trial

UK BEAM was a multi-centre and multi-arm randomised controlled trial (RCT) (ISRCTN32683578) in which 1,334 participants with non-specific low back pain were randomised. The trial methods are described in detail elsewhere; [14] but briefly, balanced randomisation was used to allocate participants to one of four different complex interventions: *best care, best care plus an exercise programme, best care plus a spinal manipulation package, or a combined treatment package of best care, exercise, and spinal manipulation*. The primary outcome measure used in the trial was the Roland Morris Disability Questionnaire (RMDQ). The RMDQ is a 24-item validated questionnaire measuring disability and is one of the most commonly cited and used PROMs in studies of back pain. [15]–[17] Scores on the RMDQ range from 0 to 24, where higher scores indicate greater disability. The score is derived from the sum of the specific item statements that a participant agrees with; an example statement is ‘Because of my back, I use a handrail to get upstairs’. A secondary outcome in the trial was the modified von Korff Scales, which measure chronic pain and disability due to LBP. [18] The scores are calculated from responses to three questions each for pain and disability; an example question from this questionnaire is ‘In the past 4 weeks how much has your back pain interfered with your daily activities?’. Participants then respond using a numerical rating that is graded from 0 to 10, where zero is none and 10 is ‘*unable to do any at all*’, ‘*extreme*’, or ‘*as bad as it could be*’, depending on the question. The average of the three numerical rating scales for each domain are taken and multiplied by 10. [18] The outcome measures in UK BEAM were completed at baseline, one month, six months, and then finally at one year. At each follow-up time point, participants also completed a health transition question, which was included with the questionnaires. This is a single question assessing global perception of change, by asking participants whether they have experienced improvement or deterioration in their low back pain since beginning treatment. [19] It has seven possible responses ranging from ‘*completely recovered*’ to ‘*vastly worsened*’.

### Use of data from the UK BEAM trial

We dichotomised the health transition scale as suggested by Lauridsen *et al* (and as is typical), categorising patients as improved if they were in the top two categories of recovery, using this as our external criterion for generating ROC curves. [20] We then purposively sampled RMDQ data, and data from the modified von Korff disability scale, across all trial arms, and from the Best Care arm in particular, at three-months, and one-year follow-up time points.

### Ethics statement

The trial protocol was approved by the Northern and Yorkshire multi-centre research ethics committee and 41 local research ethics committees. No additional ethics approval was required to reuse the anonymous trial data in this analysis.

## Results and Discussion

**Figure 1:**
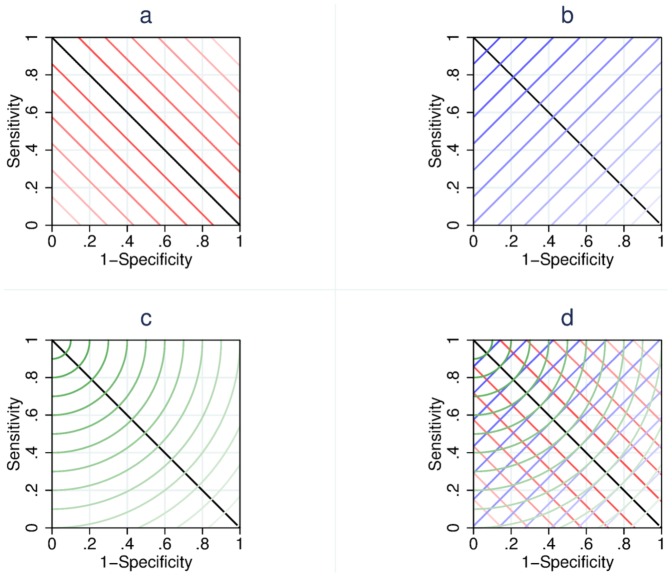
Contour diagrams. The figure shows the contour diagrams we constructed for the Farrar estimator (Figure 1a), the EMGO estimator 2 (Figure 1b), and the sums of squares estimator (Figure 1c), and contours resulting from all three estimators in the same space (Figure 1d).

Figure 1 shows the contours of the quantities being minimised by each of the estimators we explored; estimator 1, the Farrar method (Figure 1a); estimator 2, the EMGO method (Figure 1b); estimator 3, the sum of squares method (Figure 1c), and contours resulting from all three estimators in the same space (Figure 1d). In each of these figures the contour lines describe the points within the ROC space that are valued equally by the estimator. The intensity of the line indicates the magnitude of the quantity being minimised, with darker lines indicating preferential selection for MIC.

In Figure 1a, the contours show how the Farrar estimator chooses the cut-point that falls closest to the 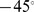 tangent line (*i.e.* the black line passing from (1,0) to (0,1)). The cut-point falling closest to this line, *i.e* that point which is intersected by the darkest (reddest) contour line, will be chosen using this method. Figure 1b shows how, in contrast, using the the EMGO method the point which is chosen is that point which is intersected by the darkest (bluest) diagonal line that runs orthogonally to the 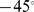 tangent line. Finally, our sums of squares method is illustrated in Figure 1c where the contours are formed of circle arc segments centred around (1,0) and the point which is intersected by the darkest (greenest) contour is selected. We note that, in each case, an infinite number of lines exist and we have rendered only some of these for the sake of clarity.

**Figure 2:**
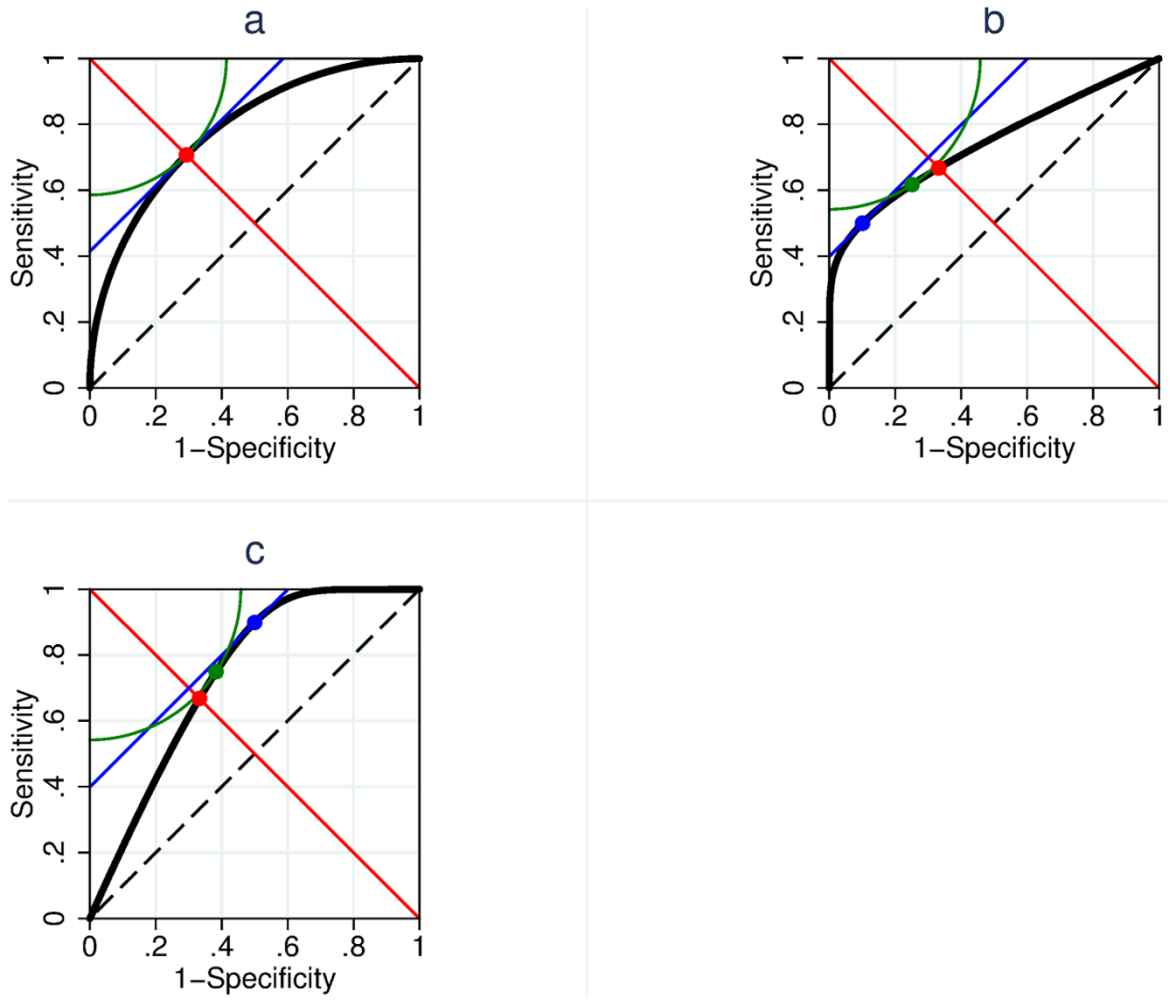
Hypothetical plots. The figure shows three hypothetical examples of well-behaved ROC curves when the number of cut-points is large (*i.e.* tending toward a continuous nature). Panel 2a shows a case of symmetry and a monotonically decreasing gradient where all three estimators provide the same cut-point. Panels 2b and 2c shows how the methods choose different points when there is asymmetry skewing the curve left or right.

These contour plots show that the three methods are not equivalent. In particular they demonstrate that different points of the contours drawn by EMGO method (Figure 1b) are not equidistant from the top-left corner of ROC space, but that each possible point on contours described by the sum of squares method is equidistant from the top-left corner. Exactly which point on the ROC curve is chosen depends on the positions of the cut-points in ROC space. This is more clearly demonstrated using our hypothetical plots in Figure 2.

### Hypothetical examples

Figure 2 shows three hypothetical examples of well-behaved (*vide supra*) ROC curves when the number of cut-points is large (*i.e.* tending toward a truly continuous nature). Figure 2a shows a case in which all three estimators provide the same cut-point; we note for later discussion that away from the point of convergence, divergence happens quickly between the sums of squares method and the EMGO method. This example is a special case in which the ROC curve is symmetrical. When symmetry is present (and the gradient of the ROC curve monotonically decreases) all three estimators will choose the same point (even when the curve is not continuous). Conversely, if there is asymmetry skewing the curve left or right, as is the case in Figure 2b or 2c, the methods choose different points. The mechanism for this becomes clear if one considers the contours and minimum quantities chosen by each of the three methods, which are also shown in Figure 2. It is quite clear that in Figures 2b and 2c that the points chosen by both the EMGO and Farrar methods are further from the top-left corner of the ROC space than the point chosen by the sum of squares method (in so far as the blue and red points are further from the top left corner than the green contour). In the well-behaved continuous situation, such as is shown here, the point selected by the sum of squares method will always be between the points selected by the other two methods.

### Results from UK BEAM trial data

**Figure 3:**
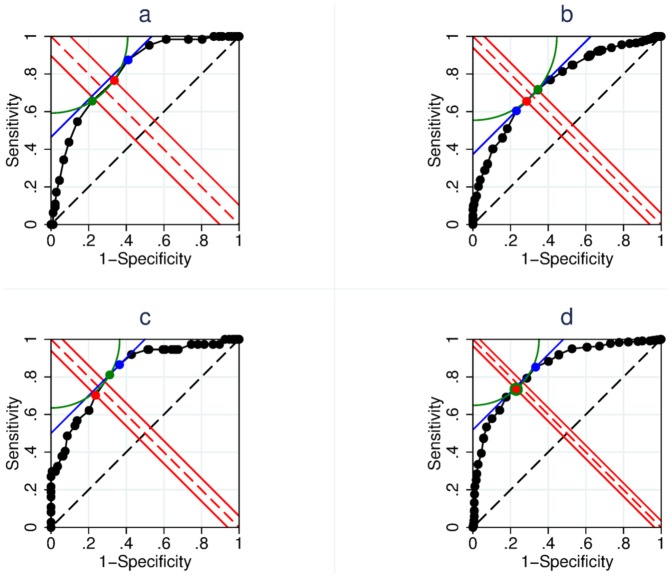
ROC curves constructed using UK BEAM trial data. The figure shows ROC curves constructed using real trial data. Panel 3a shows the Roland Morris Disability Questionnaire changes in the Best Care arm of the UK BEAM trial, at one year. The three estimators choose different points (Farrar = red, EMGO = blue, and sums of squares = green). Panel 3b shows the modified von Korff disability changes across all four arms of the trial, at three months. Panel 3c shows modified von Korff disability data from the Best Care arm at three months. Panel 3d shows modified von Korff disability data from all four trial arms at one-year.

In real applications, ROC curves often consist of a finite number of possible cut-points, rather than being continuous, and are noisy, rather than well behaved. These issues add further complexities to the selection of MIC using the different approaches. Figure 3a, comprises more typical quasi-continuous data from the RMDQ score change in the Best Care arm of the UK BEAM trial at the one-year follow-up time point. The differing points of minimisation are shown to fall on the different contours described by the different estimators. The sum of squares method corresponds to a cut-point of five points (left/green) on the 24-point scale, the Farrar method corresponds to a cut-point of four points (centre/red), and the EMGO method corresponds to a cut-point of three points (right/blue). For this example, we have provided sensitivity and specificity data in Table 1.

**Table 1:** Sensitivity and specificity data for the ROC curve in Figure 3a.

Cut-point	Sensitivity (%)	Specificity (%)	|Farrar|x100%	|EMGO|x100%	|Sum of Squares|x100%
−11	100	0	100	100	100
−10	100	0.560	99.44	99.44	99.44
−9	100	2.250	97.75	97.75	97.75
−7	100	3.930	96.07	96.07	96.07
−5	100	5.620	94.38	94.38	94.38
−4	100	8.990	91.01	91.01	91.01
−3	100	10.67	89.33	89.33	89.33
−2	100	13.48	86.52	86.52	86.52
−1	98.44	19.66	78.78	81.90	80.36
0	98.44	26.97	71.47	74.59	73.05
1	98.44	38.76	59.68	62.80	61.26
2	95.31	47.75	47.56	56.94	52.46
3†	87.50	58.99	28.51	53.51	42.87
4‡	76.56	66.29	10.27	57.15	41.06
5*	65.63	78.09	12.46	56.28	40.76
6	54.69	85.96	31.27	59.35	47.44
7	43.75	90.45	46.70	65.80	57.05
8	34.38	93.26	58.88	72.36	65.97
9	23.44	95.51	72.07	81.05	76.69
10	17.19	97.19	80.00	85.62	82.86
11	10.94	97.75	86.81	91.31	89.09
12	9.380	97.75	88.37	92.87	90.65
13	6.250	98.88	92.63	94.87	93.76
15	0	98.88	98.88	101.1	100.0

† Cut-point chosen by the EMGO method.

‡ Cut-point chosen by the Farrar method.

^*^ Cut-point chosen by the sum of squares method.

In Figure 3b, the plot was formed using modified von Korff disability data from all four trial arms in UK BEAM at three-months. Even though, on the face of it, this curve appears to be reasonably symmetrical, because there are many potential cut-points, even relatively subtle differences are minimised differently by the contours described by the different estimators. In this case, the sum of squares method favours a cut-point of 14.333 (*NB* thirds are common in modified von Korff scores, which is based on an average of three ratings; right/green), the Farrar method a cut-point of 17.667 (centre/red), and the EMGO method a cut-point of 21.000 points (left/blue).

Figure 3c shows modified von Korff disability data from the Best Care arm at three-months. In this example, the sum of squares method favours a cut-point of 14.333 (centre/green), the Farrar method favours a cut-point of 17.667 (left/red), and the EMGO method favours a cut-point of 11.000 (right/blue).

Finally, Figure 3d shows modified von Korff disability data from all four trial arms arm at one-year. Both the sum of squares method and the Farrar method estimates a cut-point of 21.000 (left/green), but the EMGO method favours a cut-point of 14.333 (right/blue).

## Considerations

The results demonstrate that the three estimators can lead to different cut-points and that, in practice, the differences can be magnitudes of several points. This has implications for interpreting improvement in individual patients, and for interpreting the number or proportion of improved patients in clinical trial arms. [21] In responder analyses, (*i.e.* analyses involving comparisons of the number of individual improvements in trial arms), the choice of method could affect trial results and therefore trial conclusions. We note that when conducting responder analyses the minimal detectable change of the outcome measure must also be examined, but we consider a detailed discussion of this to be outside the scope of this paper and on this point refer the reader to other material. [5], [8]

The Farrar method finds the point at which sensitivity and specificity are closest together. One problem with this approach is that while ROC curves are monotonic in nature, it is possible for the shape of curve to be such that it crosses the 45 degree tangent line at a point where sensitivity and specificity are closest together, but with a combination of sensitivity and specificity that is less appealing (*i.e*. further from the top-left corner) than another point on the ROC curve. While the EMGO approach avoids this weakness, it can produce cut-points that are less sensible in terms of disproportionately minimising either sensitivity or specificity, when the minimising cut-point is at a lateral extreme of a contour described by its estimator. The EMGO cut-point is sometimes described as the point for which the sum of the percentages of misclassified patients is lowest, [7] and the sum of 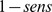 and 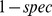 as the proportion of misclassification. [10] However, we suggest that it is not correct to describe the approach in this way, as the percentage of misclassified patients is dependent on the prevalence of improvement and is calculated as one minus sensitivity, multiplied by the total number of improved patients, plus the specificity multiplied by the total number of non-improved patients.

Our sums of squares approach always selects the cut-point closest to the top-left corner (1,0) of ROC space. Furthermore, even in the situation where a ROC curve is approximately symmetrical and all three methods should choose the same point, the EMGO method will be more sensitive to noise than the either the sum of squares or Farrar method, and as such should be avoided. This can be seen from Figure 2a, where at the point of convergence, the contour described by our method and the contour described by the EMGO method are both adjacent to the ROC curve. Following the path of the contours, one can see that the contour described by our method diverges away from the curve, in contrast to the EMGO method that sits on the curve, in what could be a noisy zone. For this reason the estimator we propose is more efficient.

Finally, we acknowledge that our proposed approach and its utility is obvious from simple Euclidean geometry and as such this presented work should be unnecessary. Nevertheless, since currently non-optimal approaches are in widespread use, we considered that this study was necessary to provide clarification and to highlight the extent to which different approaches can affect results. We urge epidemiologists to more carefully consider cut-point selection when using ROC curves to make decisions about MIC thresholds. To help with this, we provide a Stata module that produces estimates based on all three of the approaches discussed, along with a help file, which is held in the RePEc Statistical Software Components archive, and may be installed at the Stata prompt by typing *ssc install rocmic*. [22]
