# Correction: Using ROC Curves to Choose Minimally Important Change Thresholds when Sensitivity and Specificity Are Valued Equally: The Forgotten Lesson of Pythagoras. Theoretical Considerations and an Example Application of Change in Health Status

**DOI:** 10.1371/journal.pone.0120967

**Published:** 2015-03-16

**Authors:** The PLOS ONE Staff

There is an error in affiliation 2 for author Robert Froud. Affiliation 2 should be: Norges Helsehøyskole, Campus Kristiania, Prinsens Gate 7-9, Oslo, Norway.

In Table 1, the Sum of Squares x100% column is incorrect. Please see the corrected Table 1 here.

**Table 1:** Sensitivity and specificity data for the ROC curve in Fig. 3A

Cut-point	Sensitivity (%)	Specificity (%)	|Farrar|x100%	EMGO x100%	Sum of Squares x100%
−11	100	0	100	100	100
−10	100	0.560	99.44	99.44	98.88
−9	100	2.250	97.75	97.75	95.55
−7	100	3.930	96.07	96.07	92.29
−5	100	5.620	94.38	94.38	89.08
−4	100	8.990	91.01	91.01	82.83
−3	100	10.67	89.33	89.33	79.80
−2	100	13.48	86.52	86.52	74.86
−1	98.44	19.66	78.78	81.90	64.57
0	98.44	26.97	71.47	74.59	53.36
1	98.44	38.76	59.68	62.80	37.53
2	95.31	47.75	47.56	56.94	27.52
3 †	87.50	58.99	28.51	53.51	18.38
4 ‡	76.56	66.29	10.27	57.15	16.86
5*	65.63	78.09	12.46	56.28	16.61
6	54.69	85.96	31.27	59.35	22.50
7	43.75	90.45	46.70	65.80	32.55
8	34.38	93.26	58.88	72.36	43.51
9	23.44	95.51	72.07	81.05	58.82
10	17.19	97.19	80.00	85.62	68.65
11	10.94	97.75	86.81	91.31	79.37
12	9.380	97.75	88.37	92.87	82.17
13	6.250	98.88	92.63	94.87	87.90
15	0	98.88	98.88	101.12	100.01

† Cut-point chosen by the EMGO method

‡ Cut-point chosen by the Farrar method

* Cut-point chosen by the sum of squares method
